# Refinement of *Bos taurus *sequence assembly based on BAC-FISH experiments

**DOI:** 10.1186/1471-2164-12-639

**Published:** 2011-12-30

**Authors:** Giulia Partipilo, Pietro D'Addabbo, Giovanni M Lacalandra, George E Liu, Mariano Rocchi

**Affiliations:** 1 Department of Biology, University of Bari, Via Orabona 4, 70125 Bari, Italy; 2 Department of Animal Production, Faculty of Veterinary Medicine, University of Bari, SP. Casamassima-Valenzano, Valenzano, BA 70010, Italy; 3 Bovine Functional Genomics Laboratory, U.S. Department of Agriculture-Agricultural Research Service, Beltsville, MD 20705, USA

**Keywords:** Cow genome, alternate assemblies of cow genomes, genomic comparison, unassigned scaffolds, BAC-FISH mapping

## Abstract

**Background:**

The sequencing of the cow genome was recently published (Btau_4.0 assembly). A second, alternate cow genome assembly (UMD2), based on the same raw sequence data, was also published. The two assemblies have been subsequently updated to Btau_4.2 and UMD3.1, respectively.

**Results:**

We compared the Btau_4.2 and UMD3.1 alternate assemblies. Inconsistencies were grouped into three main categories: (i) DNA segments showing almost coincidental chromosomal mapping but discordant orientation (inversions); (ii) DNA segments showing a discordant map position along the same chromosome; and (iii) sequences present in one chromosomal assembly but absent in the corresponding chromosome of the other assembly. The latter category mainly consisted of large amounts of scaffolds that were unassigned in Btau_4.2 but successfully mapped in UMD3.1. We sampled 70 inconsistencies and identified appropriate cow BACs for each of them. These clones were then utilized in FISH experiments on cow metaphase or interphase nuclei in order to disambiguate the discrepancies. In almost all instances the FISH results agreed with the UMD3.1 assembly. Occasionally, however, the mapping data of both assemblies were discordant with the FISH results.

**Conclusions:**

Our work demonstrates how FISH, which is assembly independent, can be efficiently used to solve assembly problems frequently encountered using the shotgun approach.

## Background

Many genomes have been sequenced using the whole-genome shotgun method, in which the sequence assembly is prepared from short, unmapped sequence reads with the help of specific software. The presence of interspersed repeats and segmental duplications in eukaryotic genomes poses serious challenges to genome assembly. The end sequencing of genomic BAC clones (BAC end sequence, BES), as well as marker order data provided by linkage and/or radiation hybrid maps, has been used to guide assembly work and discriminate alternative assembly hypotheses. A correct assembly, indeed, has important implications in understanding genome organization and evolution, deciphering long-distance gene regulation, avoiding misinterpretation of polymorphisms, identifying pathologies directly or indirectly linked to features of genome architecture, and interpreting the 3D reconstruction of interphase nuclei [1-4].

The sequence assembly of the cow (*Bos taurus*) genome (Btau_4.0), similar to the genomes of rat and sea urchin [5,6], was achieved by using an intermediate approach. A number of BACs were sequenced to aid and resolve assembly problems, with special consideration to uncertainties stemming from the occasional difference between the two haploid sets [7,8]. Using the same pool of raw sequence data but different bioinformatics tools, Zimin and colleagues [9] published an alternative assembly of the cow genome (UMD2). The two assemblies represent a paradigmatic example of how challenging it can be to create a sequence assembly based on whole-genome shotgun methods alone [10].

We recently illustrated how molecular cytogenetics, using cohybridization FISH experiments of BAC clones (BAC-FISH), can discriminate between alternative hypotheses of synteny arrangements, orientation, and adjacencies [11]. The power of the BAC-FISH technique is based on the fact that it is assembly independent. We have recently emended, with this approach, misassembled segments up to 24 Mbp in size in the macaque genome [12]. In this paper we use the same approach and, occasionally, long-range PCR to resolve mapping discrepancies of the two cow genome assemblies.

## Results and Discussion

The cow sequence assembly released by Liu *et al*. [8] (Btau_4.0; http://www.hgsc.bcm.tmc.edu/project-species-m-Bovine.hgsc?pageLocation=Bovine), also available on the major genome browsers since 2007, was recently updated to the nearly identical Btau_4.2 release (used in this paper; Btau henceforth). The alternative assembly published by Zimin et al. (UMD2 [9]) was also refined. The last one, UMD3.1 draft (UMD henceforth; ftp://ftp.cbcb.umd.edu/pub/data/assembly/Bos_taurus/Bos_taurus_UMD_3.1/) is the version we used in the present study. Only recently it was included in UCSC and Ensembl browsers.

The main difference between the Btau and UMD assemblies was a substantially larger amount of unassigned sequences (ChrUns) present in Btau (11, 895 ChrUns, up to 3.5 Mbp in size, for a total of about 283.5 Mbp) compared to UMD (3, 286 ChrUns, up to 180 kbp, for a total of about 9.8 Mbp).

### Bioinformatics identification of Btau/UMD inconsistencies

**Figure 1:**
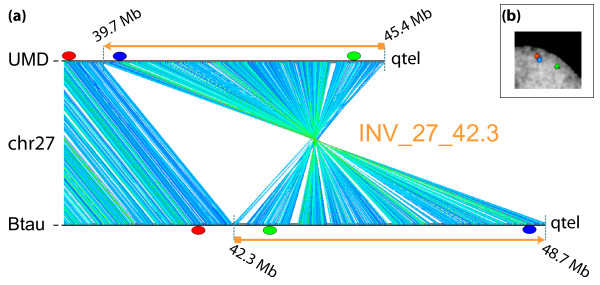
**Example of GenAlyzer analysis (INV_27_42.3)**. (a) GenAlyzer graphic output of pairwise comparisons between masked Btau (bottom line) and UMD (upper line) sequences of the chr27 subtelomeric region. The blue/green/red lines connect the matching regions. Line colors vary according to the length of the matching segment. The two orange arrows encompass the inverted region of about ~6 Mbp in size. The red/blue/green ovals indicate the map position of the BACs selected to perform FISH experiments in interphase nuclei, an example of which is shown in (b). The signal arrangement clearly supports the UMD.

The Btau and UMD masked sequences of each *Bos taurus *chromosome were first compared using the GenAlyzer software [13]. The graphic output allows to easily identify the following inconsistencies in DNA segments: (i) almost coincidental chromosomal mapping but discordant orientation (INVersions, INVs; see example in Figure 1a); (ii) Mapping in Distinct Positions (MDPs) along the chromosome; and (iii) present only in one chromosome but absent in the corresponding chromosome of the other assembly (One-Draft only Sequences, ODSs). For easy identification of inconsistent sequence assembly, we assigned to these sequences a code composed of the category they belong to followed by the chromosome number and the sequence position (in kbp).

Inconsistencies larger than 100 kbp revealed by the bioinformatics analyses are reported in the Additional Files 1, 2, 3, 4, 5, 6 and 7. Shorter segments were not considered in the present study. The largest block was an inversion spanning 6.4 Mbp on Btau (INV_27_42.3; Figure 1a). The vast majority of the inconsistencies were represented by the ODSs.

In summary, we found 135 INVs (Additional File 1), 16 MDPs (Additional File 2), and 235 ODSs (Additional Files 3 and 4). 198 ODSs were found in UMD (UMD ODSs) while only 37 in the Btau assembly (Btau ODSs). These are listed in the Additional Files 3 and 4, respectively. The much lower number of Btau ODSs with respect to UMD ODSs was expected because of the consistently larger amount of ChrUns in the Btau assembly.

**Figure 2:**
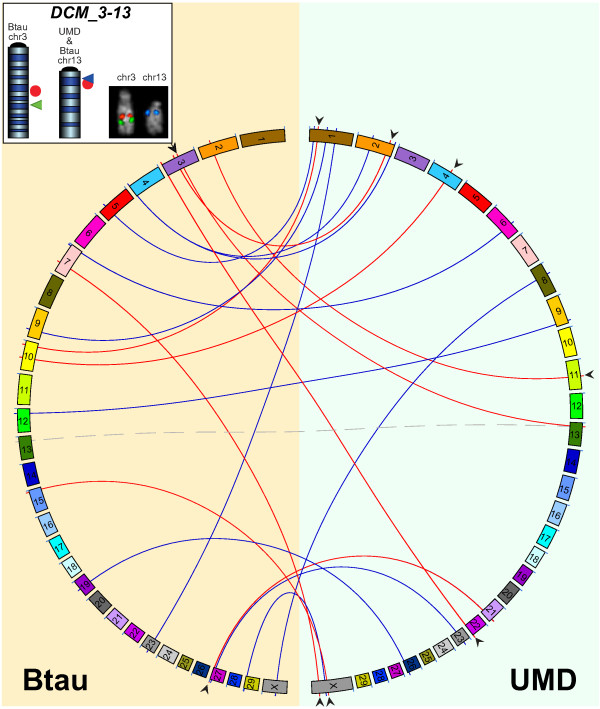
**Interchromosomal comparison between Btau and UMD**. Graphic output of Circos software illustrating all DCMs larger than 100 kbp. DCMs are depicted as lines connecting the Btau chromosomes (left side on orange background) to the UMD chromosomes (right side on light blue background). The red lines are for DCMs tested by FISH. The black arrowheads point to the assembly supported by the FISH results. In the case of the DCM_3-13, the UMD chr13 is connected to two Btau chromosomes (3 and 13) by two lines: the gray dashed line indicates that the sequence is present in the corresponding Btau chromosome; the red line points to its duplication on Btau chr3. The upper box shows chr3 and chr13 ideograms on which are reported the bioinformatics positions of BAC CH240-123I9 (internal to the DCM_3-13 sequence; red dots) according to Btau and UMD and the positions of the two chromosome-specific BACs, in green for chr3 and blue for chr13. The BAC-FISH experiment reported on the right clearly shows that the sequence is present only on chr3 (for detail see text).

All ODSs were aligned to the remaining chromosomes and unassigned scaffolds of the other assembly using megaBLAST [14] or GenAlyzer. Most of the UMD ODSs (160) aligned only to Btau ChrUn scaffolds (Additional Files 3 and 8). In just 17 cases the ODS of one assembly pointed to an ODS on the other assembly (i.e. segments mapped to different chromosomes) (Additional File 5). The paired ODSs were always flanked by non-matching DNA stretches. It could be hypothesized that the flanking non-matching sequences could be responsible for the discordant chromosomal assignments. These paired ODSs were named Discordant Chromosome Mapping sequences (DCMs; red and blue lines in Figure 2). Each DCM code report the two involved chromosomes (listed in Additional File 5). The DCM_3-13 and DCM_12-9 sequences are noteworthy. They are single copy in UMD, mapping on chromosomes 13 and 9, respectively. Btau reports a duplicate, second location for both of them, on chromosomes 3 and 12, respectively.

Some discordant segments were almost completely composed of gaps and/or repeats. These are listed in Additional Files 6 (UMD) and 7 (Btau) but were not considered in the study.

### Selection of BACs mapping in Btau/UMD discordant regions

Because of the size of the BAC clones, we took into account only MDP and DCM inconsistencies larger than 200 kbp (in bold in Additional Files 2 and 5, respectively). In the case of INVs, only segments larger than 500 kbp were considered (bold in Additional File 1), because, in order to disentangle INV cases, we needed three distinct non-overlapping BACs: one mapping inside the segment and the other two mapping outside but close to the opposite borders. The 20 inconsistencies tested by FISH are reported in Table 1 (yellow background in Additional Files 1, 2 and 5). The BAC search provided appropriate probes for 10 INVs and 2 MDPs (Additional File 9) and 9 DCMs (Additional File 10).

**Table 1:** Disambiguated INV and MDP inconsistencies

Code	Test	Supported draft
INV_1_24	FISH/int	UMD (85%)
INV_1_53.3	FISH/int	UMD (94%)
INV_4_7.2	FISH/int	UMD (90%)
INV_7_103.2	FISH/int	UMD (96%)
INV_10_59.3	FISH/int	UMD (95%)
INV_14_32	FISH/int	UMD (73%)
INV_20_0	FISH/int	UMD (98%)
INV_23_0	FISH/met.	UMD
INV_26_18.8	FISH/int	UMD (75%)
INV_27_42.3	FISH/int	UMD (90%)

MDP_4_16.2	FISH/int	Btau (98%)
MDP_5_9.6	FISH/met.	UMD
MDP_7_7.6	PCR	UMD
MDP_7_43.1	PCR	-
MDP_28_0.7	PCR	UMD

DCM_2-11	FISH/met	UMD
DCM_3-13	FISH/met	Btau
DCM_3-2	FISH/met	UMD
DCM_3-22	FISH/met	UMD
DCM_7-X	FISH/met	UMD
DCM_10-1	FISH/met	UMD
DCM_10-4	FISH/met	UMD
DCM_15-X	FISH/met	UMD
DCM_27-21	FISH/met	Btau

INV, MDP, or DCM inconsistencies (first column) disambiguated using the method reported in the second column: FISH on interphase nuclei (FISH/int), FISH on metaphases (FISH/met), or PCR. The third column indicates the draft [UMD, Btau, or neither of them (-)] which is in agreement with the experimental results. In the case of FISH performed in interphase nuclei, the round bracket reports the percentage in which the pattern of signals support the draft reported on the left of the brackets. In all cases p(H_0_) was < 0.0004%.

All UMD ChrUns were less than 200 kbp in size and were not taken into account. For the Btau ChrUns, appropriate BAC clones were searched and identified for the 50 largest ChrUns (size range: 0.5 - 3.5 Mbp), reported in Additional File 11. To simplify the reporting of these BACs in other tables and figures, we assigned a code to each BAC (third column of Additional File 11). Most of these BACs were entirely or almost entirely mapped in UMD to a single locus. For these segments we successfully searched a single BAC mapping within the scaffold (Additional File 11). In some cases, different portions of the same scaffold mapped to distinct loci. In these cases we searched for two distinct appropriate BACs. The search was successful for scaffolds 004.3, 004.10, 004.11, 004.22, 004.35, and 004.39 (Additional File 11). Portions of the Btau unassigned scaffolds 004.35 and 004.39 mapped to multiple sites of UMD chromosomes (Additional File 11).

All cow autosomes are acrocentric and difficult to distinguish on the basis of DAPI banding. Therefore, we assembled a panel of chromosome-specific BACs, one for each autosome and one for chromosome × (chrX), mapping in Btau/UMD highly concordant regions, to be used as a reference (Additional File 12). Each yielded a FISH signal consistent with its sequence position along the chromosome.

### Results of FISH experiments

The BAC-FISH approach comes with a caveat: sequences shorter than 5-10 kbp are not visualized by FISH. As a consequence, small deletions and duplications can be missed. Additionally, although the cell line used in this study (AG08501, see Methods) belongs to the same Hereford cattle breed of Dominette (used for sequencing [8]), inter-individual differences in copy number variation can bias data comparisons. It is also worth noting that the BAC library RP42 was derived from a bull of a different strain (Holstein). However, array-CGH experiments, performed to compare Dominette and AG08501, showed only two small variations (< 8 kbp sequence loss) internal to two of the BACs used to disambiguate inconsistencies. As a consequence, this bias should not affect our results (GL, personal communication).

**Figure 3:**
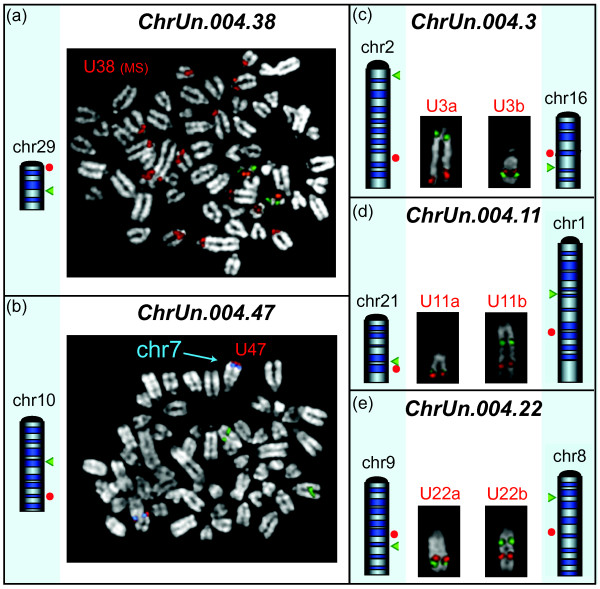
**Examples of FISH experiments of five unassigned Btau scaffolds**. The red dots beside the ideograms graphically indicate the mapping, in UMD, of the sequence spanned by the BACs selected for the Btau ChrUn under study (Additional Files 8 and 11). The green arrowheads indicate the position of the chromosome-specific reference BACs. The FISH images show the results of (a) BAC U38 producing multiple signals in disagreement with the UMD single position on chr29 and (b) BAC U47 yielding signals on chr7 and not on chr10 as reported in UMD. (c, d, e) FISH results shown for BACs U3a/U3b (ChrUn 004.3), U11a/U11b (ChrUn 004.11), and U22a/U22b (ChrUn 004.22) supporting the UMD mapping (for detail see text).

**Figure 4:**
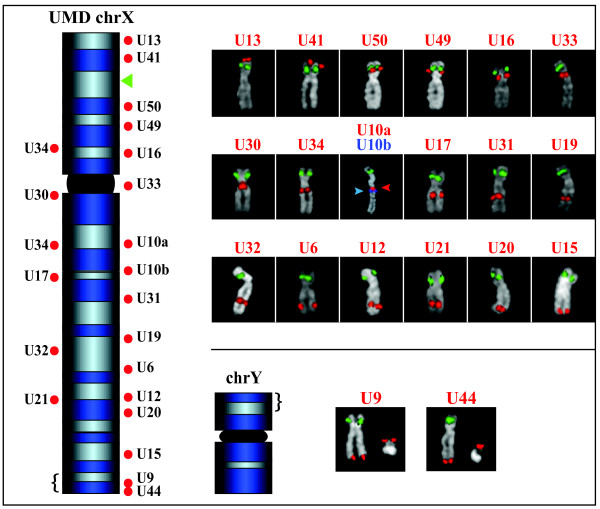
**FISH results of the sampled ChrUn scaffolds mapped in UMD to chrX**. Red dots close to the chrX ideogram graphically show the bioinformatics position of 21 BACs specific for the Btau unassigned scaffolds, according to the UMD (Additional Files 8 and 11). Two distinct portions of ChrUn 004.10 were mapped on UMD to two distinct, close regions of chrX (U10a and U10b in the ideogram). The green arrowhead represents the position of the reference BAC. All FISH results are in agreement with the UMD mapping, except U34 yielding only one signal (see text). BACs U9 and U44 yield signals on both chrX and chrY because they map to the pseudoautosomal region (brace) of UMD chrX (see text for details).

The consistency between the FISH signal position of a BAC clone with respect to the location of its sequence on the chromosome assembly was assessed by visual inspection (see Figures 3 and 4). In all cases the FISH signals were either perfectly compatible with the bioinformatics data or completely discordant (mapping, for instance, to a different chromosome).

### INV inconsistencies

Inversions are the largest discrepancies between Btau and UMD assembled chromosomes (see Figure 1; see also Figure 3 in [9]). FISH experiments were able to disambiguate all 10 INVs for which appropriate BACs were identified. In all these INV cases the results were in agreement with the UMD assembly (Table 1). Figure 1b shows the FISH results on the largest inversion, about 6 Mbp in size, present on chr27.

### MDP inconsistencies

Appropriate BAC clones were identified for two MDPs (MDP_5_9.6 and MDP_4_16.2; Additional Files 2 and 9). The FISH results supported UMD in the case of MDP_5_9.6 and Btau in the second case. The mapping position distances of the other MDPs were too small to be disambiguated by FISH. The disambiguation of two additional MDPs (MDP_7_7.6 and MDP_28_0.7) was then achieved using PCR experiments. In both cases the PCR experiments clearly supported the UMD assembly (Table 1). The same PCR approach was attempted to disambiguate the MDP_7_43.1 mapping, but all primer combinations gave negative results, thus suggesting that both assemblies could be incorrect. Primers used in PCR experiments are listed in Additional File 13.

### Btau ChrUn/UMD ODS inconsistencies

As mentioned, we sampled the 50 largest Btau ChrUns. We first tested using FISH the 33 Btau ChrUns mapped entirely to a single locus in UMD (see above; Table 2; Additional Files 8 and 11). Each BAC was cohybridized with the appropriate reference BAC for chromosome identification (Additional File 12). The FISH experiments perfectly matched the UMD mapping data (Additional File 14) in all cases except ChrUns 004.38 and 004.47. The 004.38 scaffold was found to map to a single locus close to the centromere on chr29 in UMD (Additional File 8). The related BAC CH20-439M7 yielded a major signal on the pericentromeric region of chr29 in agreement with the UMD data but also yielded tiny signals on several other pericentromeric regions (Figure 3a). Pericentromeric regions are well known to harbor segmental duplications frequently shared with other pericentromeric regions [15].

**Table 2:** Bioinformatic and experimental mapping of 50 largest Btau ChrUns

ChrUn	BAC code	UMD	FISH	ChrUn	BAC code	UMD	FISH
004.1	U1	14	14	004.29	U29	1	1
004.2	U2	16	16	004.30	U30	X	Xq
004.3	U3a	2	2	004.31	U31	X	Xq
	U3b	16	16	004.32	U32	X	Xq
004.4	U4	12	12	004.33	U33	X	Xq
004.5	U5	12	12	004.34	U34	X	Xq
		21 *		004.35	U35a	2	2
		20 *			U35b	4	MS
004.6	U6	X	X			22	
004.7	U7	7	7			26	
004.8	U8	13	13			28	
004.9	U9	X	Xq, Y	004.36	U36	19	19
004.10	U10a	X	Xq	004.37	U37	7	7
	U10b	X	Xq	004.38	U38	29	**29, MS**
004.11	U11a	21	21	004.39	U39a	15	**23**
	U11b	1	1		U39b	23	MS
004.12	U12	X	Xq			24	
004.13	U13	X	Xp			21	
004.14	U14	6	6			14	
004.15	U15	X	Xp			22	
004.16	U16	X	Xp			20	
004.17	U17	X	Xq			22	
004.18	U18	18	18	004.40	U40	3	3
004.19	U19	X	Xq	004.41	U41	X	Xp
004.20	U20	X	Xq	004.42	U42	U	**8**
004.21	U21	X	Xq	004.43	U43	12	12
004.22	U22a	9	9	004.44	U44	X	Xq, Y
	U22b	8	8	004.45	U45	21	21
004.23	U23	12	12	004.46	U46	5	5
004.24	U24	21	21	004.47	U47	10	**7**
004.25	U25	3	3	004.48	U48	U	**8**
004.26	U26	15	15	004.49	U49	X	Xp
004.27	U27	12	12	004.50	U50	X	Xp
				
004.28	U28	15	15				

BACs mapping within the 50 largest Btau ChrUns were hybridized in metaphase nuclei. Cases where the bioinformatic and biological position were discordant are in bold.

The Btau ChrUn 004.47 mapped to UMD chr10 (Additional File 8). Contrary to this mapping, the related BAC CH240-0025K7 (U47 in Additional File 11 and Figure 3b) gave a signal on the subtelomeric region of chr7 (Figure 3b).

**Figure 5:**
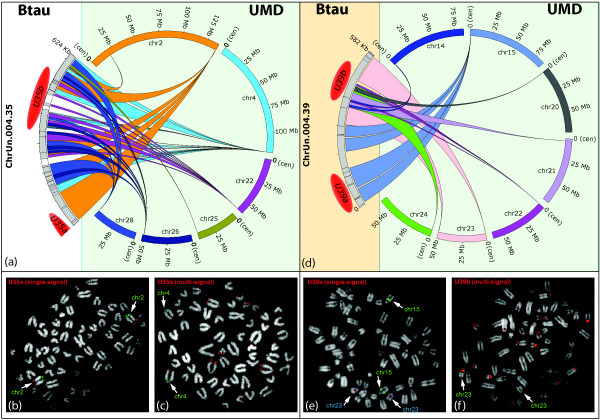
**Mapping of the ChrUns 004.35 and 004.39**. The complex mapping on UMD of sequences composing the Btau unassigned scaffolds 004.35 and 004.39 is illustrated by a Circos graphic output in (a) and (d), respectively. The red ovals indicate the bioinformatics mapping of the BACs used in the FISH experiments, results of which are reported in the boxes below. The half-oval indicates that the mapping of the BAC U35a was inferred by the matching of a single BES. The second BES was found on chr2 in both drafts. (b) FISH results of BAC U35a confirming its unique localization on chr2. (c) Multiple signals yielded by BAC U35b (see Circos graphics in 5a). (e) FISH results of BAC U39a (red) cohybridized with chr15- and chr23-reference BACs (green and blue spots, respectively) showing that it maps on chr23 and not on chr15. (f) Multiple FISH signals produced by BAC U39b (see text for details).

We then performed FISH experiments to clarify the mapping of Btau ChrUns that UMD assigned to two or more loci (Table 2; Additional Files 8 and 11). Scaffolds 004.3, 004.11, and 004.22 split into two domains mapping to different chromosomes (Additional File 8) in UMD; ChrUn 004.10 split into two distinct regions of chrX (U10a and U10b in Additional File 8). FISH experiments using BACs specific for each domain were in perfect agreement with the UMD mapping (Additional Files 11; Figures 3c-e and 4). A large part of Btau unassigned scaffold 004.5 was mapped to chr12, with the exception of two distinct 17 kbp and 27 kbp DNA stretches that UMD duplicated to chr20 and chr21, respectively (Additional File 8). These duplications were not detected by FISH, suggesting that the duplications could be assembly mistakes. GenAlyzer analysis unveiled a more complex situation for the two Btau unassigned scaffolds 004.35 and 004.39 (Figure 5 and Additional File 8). The 5' portion of the ChrUn 004.35 (117 kbp) mapped to the subtelomeric region of chr2. The rest of it was composed of sequence stretches duplicated to six distinct UMD subtelomeric regions (chromosomes 2, 4, 22, 24, 25, and 26; Figure 5a). Our *in silico *BAC screening identified two clones: U35a and U35b. The first BAC confirmed the unequivocal mapping of the 5' sequence stretch of ChrUn 004.35 to chr2 (Figure 5b). The second BAC, in addition to the six subtelomeric positions predicted in UMD, disclosed six additional subtelomeric signals (Figure 5c). Low-copy repeats scattered over pericentromeric and subtelomeric regions are a frequent finding in mammals [16,17]. The complex mapping of the ChrUn 004.39 in UMD is summarized in Figure 5d. The BAC U39a, covering the region that UMD unequivocally mapped to chr15 (Figure 5c), clearly indicated that instead this domain maps to chr23 (Figure 5e). The BAC U39b, covering the region scattered to seven different loci in UMD assembly (centromeric regions of chromosomes 14, 20, 21, 22, 23, and 24, and telomeric region of chromosome 23; Figure 5d), produced FISH signals on the pericentromeric region of at least 14 chromosomes (Figure 5f). For the remaining eight Btau UMD ChrUns, which mapped to multiple loci, BLAST analysis identified only a single BAC (see above; Table 2; Additional Files 8 and 11). FISH experiments were in agreement with UMD data on the region spanned by the BAC. The only exception was U34. One BES of this BAC was mapped to Xq with the second to Xp (Additional File 11). The FISH experiment yielded signals only on Xq (Figure 4).

Finally, we performed FISH experiments, using the appropriate BACs, to map the two unassigned Btau scaffolds, 004.42 and 004.48, that were not mapped in UMD (Table 2; Additional Files 8 and 11). The FISH results mapped both of these scaffolds to the chr8 subtelomeric region.

These overall results indicate that Btau_4.2 occasionally failed to incorporate multiple copies of a unique sequence into the assembly likely due to problems posed by the complexity of some regions --pericentromeric and subtelomeric regions in particular-- and that UMD 3.0 collapsed duplications into a single copy. CH240_439M7 (U38) is an example of the latter situation, suggesting duplications may be over-collapsed in UMD3. Differences due to copy number variations were considered in this context (see caveat above).

### Btau ODS pointing to UMD ODS (DCM)

We disambiguated the nine DCM segments (yellow background in Additional File 5; red lines in Figure 2), which were larger than 200 kbp (see above). The FISH experiments, using appropriate BACs (Additional File 10), were in agreement with UMD in seven out of nine cases (Table 1; see arrowheads in Figure 2). In the DCM_27-21 case the FISH analysis was in agreement with Btau; the DCM_3-13 case, however, was peculiar. The latter sequence was mapped to chr13 in both Btau and UMD (dashed gray line in Figure 2). A duplicated copy of this sequence is present on chr3 in Btau (see above and Additional Files 5 and 10). This latter Btau mapping was the only one supported by the specific BAC clone (box in Figure 2). No hints of a FISH signal were observed on chr13.

### Chromosome X

ChrX is the chromosome showing the largest difference between the two assemblies, with strikingly fewer assembled sequences in Btau than UMD (88.5 Mbp versus 148.8 Mbp, respectively). It was not a surprise, therefore, that 20 out of the 50 Btau ChrUns we tested (approximately 15.9 Mbp in total) were mapped in UMD to chrX (Additional File 8). In all cases the FISH results and UMD were in perfect agreement.

Van Laere and colleagues [18] recently characterized the *Bos taurus *X/Y pseudoautosomal region. UMD places the Btau ChrUns 004.9 and 004.44 internal to the pseudoautosomal region as defined by Van Laere *et al*. [18]. In agreement with these data, the two BACs CH240_376E18 (U9) and CH240_0032O5 (U44) yielded telomeric FISH signals in both Xp and Y chromosomes (Additional File 11; Figure 4). These data will be helpful to researchers in defining the organization of chrY, which is poorly represented in both Btau and UMD.

## Conclusions

The presence, in eukaryotes, of various types of repeats and segmental duplications makes the assembly of their genome sequences challenging, especially if attained using a shotgun approach. Indeed, our bioinformatics comparison detected more than 380 instances in which DNA segments larger than 100 kbp were mapped inconsistently in the two drafts or assembled only by one of the two assemblies. In this paper we sampled a number of segments whose mapping was at variance in the two *Bos taurus *drafts. By hybridizing appropriate BAC clones on cow metaphase or interphase nuclei, we were able to disambiguate most of the tested inconsistencies. In the majority of cases our FISH results supported UMD. Additionally, we assigned some unassembled or partially misassembled sequences in both drafts to specific chromosomal regions (Btau ChrUn.004.42, ChrUn.004.48, ChrUn.004.47, ChrUn.004.39, and DCM_3-13).

We took into consideration only discrepancies between the two drafts. What about segments concordantly misassembled? The case of DCM_3-13 is paradigmatic in this respect. Both drafts mapped it to chr13. Btau, however, placed a duplicated copy of this segment on chr3. Our FISH analysis indicated that the sequence was uniquely located on the latter chromosome. If the duplication on chr3 was not present in Btau, we would have missed this point. We can reasonably suppose, however, that the assembly of this segment was problematic, thus providing a paradigmatic example of how assembly work can take advantage of the FISH approach.

A systematic bioinformatics/cytogenetics interaction guided the sequence assembly of the orangutan genome [19] which, therefore, represents a relevant example in this context. Hundreds of appropriate BAC-FISH experiments on orangutan metaphases produced detailed synteny maps of this genome. A graphic summary of this frame is reported at http://www.biologia.uniba.it/orang/ as an integral part of the orangutan sequencing project. A subset of the reported FISH experiments was specifically designed to disambiguate alternative assembly hypotheses.

Data on the karyotype evolution of the species under study, if available, may also be of help during the assembly. The orangutan chromosome 3 is a good example in this respect. The precise information on the distinct pericentric inversions this chromosome underwent in orangutan and human lineages since their divergence was crucial in precisely defining the orientation of the different synteny blocks in orangutan and allowed reconciling the very different organization of the chromosome in the two species (see the bottom of the page: http://www.biologia.uniba.it/orang/PPY/PPY_03.html).

As far as the cattle genome is concerned, the majority of our FISH results performed to disambiguate Btau/UMD inconsistencies supported UMD. This means that for a Btau/UMD discrepancy not disambiguated here, UMD has a better chance to be the correct form, but the assumption has to be biologically validated to avoid biased conclusions.

Some recent papers dealing with cattle genomics took into account the Btau assembly only. Their conclusions, therefore, could be biased. For example, Kommadath and colleagues [20] considered the region chr15:407, 162-23, 219, 582 as a single, continuous cluster of lowly expressed genes. On the contrary, UMD mapped part of it (chr15:15, 926, 700-16, 243, 800) on chrX (48, 846, 300-49, 164, 000). Our FISH experiment was in perfect agreement with UMD (see DCM_15-X in Table 1 and Additional Files 5 and 10).

Misassemblies can be very deceitful in genome-wide association studies. Bouwman *et al*. [21], for instance, used this approach to investigate the cattle genetic variation in milk fat composition primarily using the Btau data set. They used the UMD data just in case the segment was not assembled in Btau. Our bioinformatics analysis found that three of the reported regions (chr5:81, 900, 000-99, 900, 000; chr19:32, 700, 000-61, 800, 000; and chr23:26, 300, 000-31, 700, 000) were partly inconsistent between Btau and UMD (DCM_5-1, DCM_19-26, and DCM_23-1; Additional File 5). These regions were not among those tested by FISH in this study. Therefore, as stressed above, a biological validation of their mapping is mandatory before any conclusion is drawn. In another genome-wide association study, Cole *et al*. [22] mapped their significant SNPs in both assemblies. In some cases the mapping discrepancy was very clear because two different chromosomes were involved (for ARS-BFGL-NGS-317, for instance, see DCM_3-2 in Additional File 5). For the remaining SNPs it is not always apparent to the reader if the discrepancy affected the results or not. To conclude, all these studies make evident that a merge of the two assemblies into a single, agreed release of the cattle genome sequence is highly desirable, with cytogenetics playing an important role toward this goal.

## Methods

### Sequence Resources

Btau sequences (both chromosomes and ChrUns) were downloaded from ftp://ftp.hgsc.bcm.tmc.edu/pub/data/Btaurus/. The Btau chromosomes and the unassigned scaffolds used in the present study are also recorded in GenBank [ Genbank:NC_007299:NC_007320[accn]; GenBank:NC_007324:NC_007331[accn]; Genbank:DS490632:DS490681[accn] ]. UMD sequences was downloaded from ftp://ftp.cbcb.umd.edu/pub/data/assembly/Bos_taurus/Bos_taurus_UMD_3.1/. The UMD chromosomes and unassigned scaffolds are also recorded in GenBank [ Genbank:GK000001:GK000030[accn]; Genbank:GJ057137:GJ060422[accn] ].

### Assemblies' comparison

Sequences comparison was performed by GenAlyzer [13] according to authors' instructions, or by Megablast [14]http://www.ncbi.nlm.nih.gov/staff/tao/URLAPI/megablast.html . Some results were plotted using Circos [23].

### BAC identification

BACs specific for the region under study were obtained by querying the Trace Archives database (NCBI; http://blast.ncbi.nlm.nih.gov) in search for BAC-end sequences or shotgun reads belonging to the BAC libraries RPCI42 or CH240 http://bacpac.chori.org. Library CH240 is derived from Domino, the sire of Dominette (used for sequencing [8]) while RP42 from an unrelated Holstein bull. BACs in which both BESs matched to unique sequences, in opposite orientation, and at plausible distance (50-300 Kb) were preferentially considered. For regions where BESs gave no or inconsistent results, we searched for shotgun sequence reads belonging to the same BAC.

### FISH

Cytogenetic cattle preparations and DNA belonging to the same Hereford cow strain used for the cattle genome sequencing project were obtained from the fibroblast cell line AG0851, purchased from the Coriell Institute. Co-hybridization FISH experiments were performed as previously reported [24,25]. Digital images were obtained using a Leica DMRXA2 epifluorescence microscope equipped with a cooled CCD camera (Princeton Instruments, Princeton, NJ, USA). Cy3-dCTP, Fluorescein-dCTP, Cy5-dCTP and DAPI fluorescence signals, detected with specific filters, were recorded separately as grey scale images. Pseudocoloring and merging of images were performed using Adobe Photoshop™ software.

To disambiguate the INVs and the MDPs, for each experiment we inspected signal triplets in metaphases (10 counts) or in interphase nuclei (50 counts) (Table 1).

### Long-PCR

Long-PCRs were performed using the TaKaRa LA TaqTM. The PCR conditions, for all primers were as follows: 1 min at 94°C, followed by 30 cycles (20" at 94°C and 11' at 68°C). Primers were designed with the Primer3 software v.0.4.0 http://frodo.wi.mit.edu/primer3/ (Additional file 14).

## List of abbreviations

BAC-FISH: cohybridization FISH experiments of BAC clones; BES: BAC End Sequence; Btau: Btau_4.2 assembly; UMD: UMD3.1 assembly; ChrUn: unknown chromosome, aka unassigned or unanchored scaffold; INV: inversion; MDP: mapped in distinct position; ODS: one-draft sequence; DCM: discordant chromosomes mapping sequence.

## Competing interests

The authors declare that they have no competing interests.

## Authors' contributions

GP: main researcher; PD'A: bioinformatic analysis planner and support; GML and GL: provided experimental materials; MR: planning, writing and funding. All authors read and approved the final manuscript.

## Supplementary Material

Additional file 1 **INVs larger than 100 Kb**. INVs larger than 500 Kb are in bold; yellow fields indicate INVs tested by FISH. Click here for file

Additional file 2 **DNA segments differently mapped by the Btau and UMD along the same chromosome (MDP)**. Yellow fields mark MDPs tested by FISH. All the MDPs turned out to be larger than 200 Kb. Click here for file

Additional file 3 **UMD ODSs larger than 100 Kb**. For each ODS the Table reports: the code (column 1), the chromosome (column 2), the start of the sequence (column 3), the end of the sequence (column 4) and the size on UMD (columns 5). Chromosome and corresponding insertion-point in Btau are reported in columns 6 and 7, respectively. Portions of four UMD ODSs map to different Btau chromosomes (column 8). Column 9 lists the Btau ChrUns larger than 100 Kb and substantially matching the UMD ODS. NS (Not Significant) in the latter column means that the UMD segment identified only tiny, fragmented, and dispersed matches on Btau. Noteworthy, 13 UMD ODSs yielded results both on a Btau chromosome and unassigned scaffolds. Click here for file

Additional file 4 **Btau ODSs larger than 100 Kb**. For each ODS the Table reports: the code (column 1), chromosome (column 2), start of the sequence (column 3), end of the sequence (column 4) and the size on Btau (columns 5). In columns 6 and 7, chromosome and corresponding insertion point in UMD are reported, respectively. Portions of some Btau ODS map to different UMD chromosomes (Details in column 8). NM (No Match) in column 9 means that no matches were found between Btau ODS and the five UMD ChrUns larger than 100 Kb. Click here for file

Additional file 5 **DCMs larger than 100 Kb**. Columns 6 and 11 report the Btau ODS and UMD ODS of which the DCM is part. For *DCM_3-13 *and *DCM_12-9 *sequences no UMD ODS was found (see text). Bold codes and yellow fields were for DCM larger than 200 Kb and tested by FISH, respectively. For detail see text. Click here for file

Additional file 6 **UMD inconsistencies, larger than 100 Kb, due to gaps or repeats**. Click here for file

Additional file 7 **Btau inconsistencies, larger than 100 Kb, due to gaps**. Click here for file

Additional file 8 **Comparison between Btau ChrUns and UMD chromosomes**. The Table lists the 50 largest Btau ChrUns (columns 1-5) and their mapping to UMD chromosomes (columns 6-8). Column 9 reports the corresponding UMD ODS. Red background highlights sequences that, on UMD, are unplaced ("-" in column F) or misplaced, ascertained through the FISH results using the BAC reported in column 10. Click here for file

Additional file 9 **BAC used in FISH experiment to test INVs and MDPs**. Click here for file

Additional file 10 **BAC used in FISH experiment to test DCMs**. Click here for file

Additional file 11 **BACs used to detect the map of the 50 greatest Btau chrUns**. Data on the BACs are listed in columns 1-10. Column 11 reports the FISH results for each BAC. "MS" means multiple signals. Red background highlights sequences misplaced or not assigned in UMD. Note that UMD merged all the unassigned scaffolds in a "chrU" sequence. Click here for file

Additional file 12 **Panel of chromosome-specific BACs used as a reference in FISH experiments**. See text for details. Click here for file

Additional file 13 **Primers used in the study**. Click here for file

Additional file 14 **Summary of FISH results using BACS specific to Btau ChrUns**. Each chromosome ideogram reports on the right the bioinformatic positions, according to UMD, of the BACs internal to the Btau ChrUns (red dot) (Additional file 11), whose actual mapping was tested by FISH (Table 2). In each ideogram the green arrowhead refers to the chromosome-specific reference BAC (Additional file 12). Each FISH image reports the signal of the scaffold-specific BAC (red) and of the reference BAC (green). For FISH results of BACs yielding multiple or unexpected signals, or mapping on chrX, see Figures 3, 4 and 5. Click here for file
