# Supplementary figures and images for: Refinement of Bos taurus sequence assembly based on BAC-FISH experiments
Source: BMC Genomics. 2011 Dec 30;12:639. doi: 10.1186/1471-2164-12-639 (PMC3268123; doi:10.1186/1471-2164-12-639)

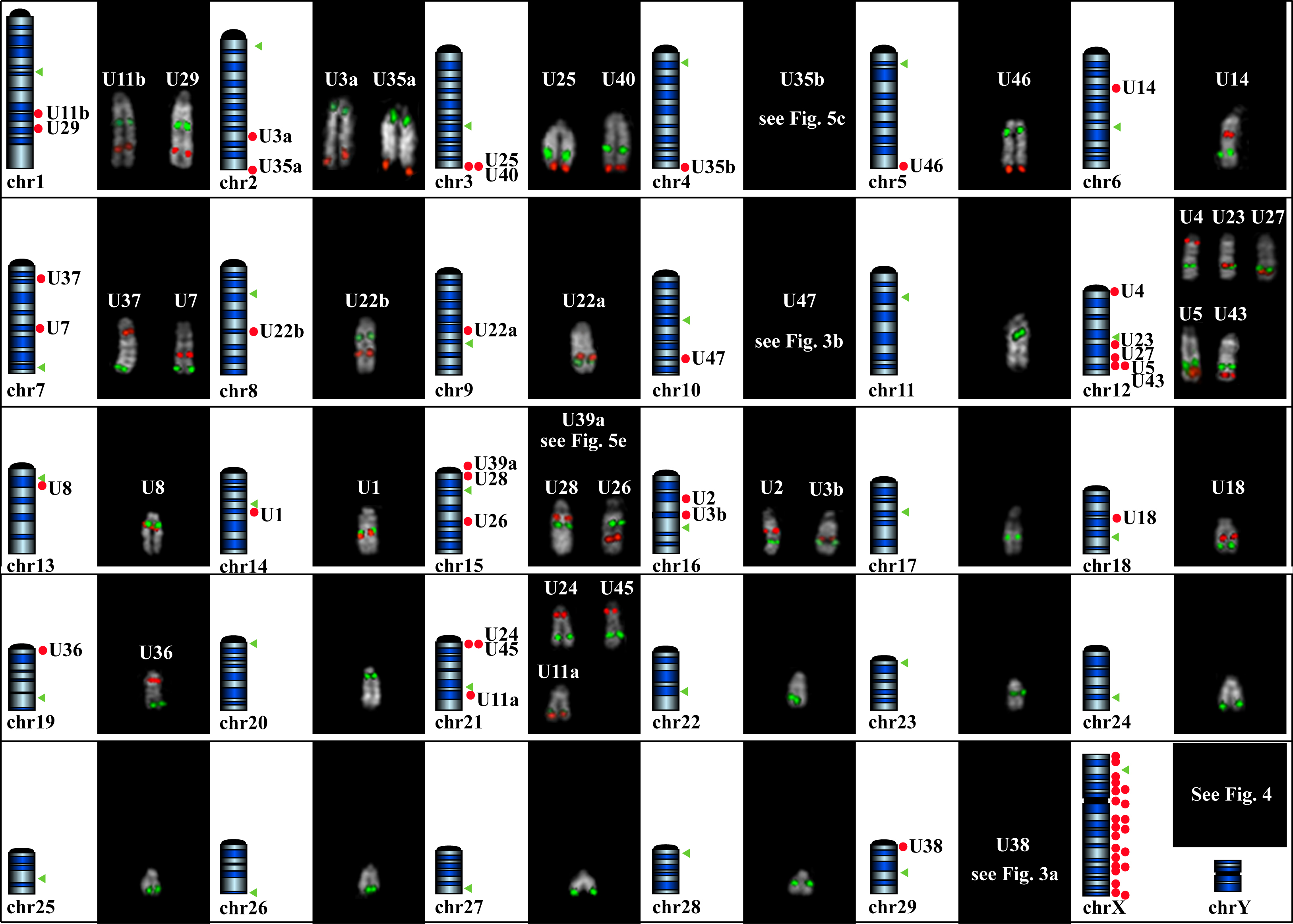

Supplement: Additional file 14 — Summary of FISH results using BACS specific to Btau ChrUns. Each chromosome ideogram reports on the right the bioinformatic positions, according to UMD, of the BACs internal to the Btau ChrUns (red dot) (Additional file 11), whose actual mapping was tested by FISH (Table 2). In each ideogram the green arrowhead refers to the chromosome-specific reference BAC (Additional file 12). Each FISH image reports the signal of the scaffold-specific BAC (red) and of the reference BAC (green). For FISH results of BACs yielding multiple or unexpected signals, or mapping on chrX, see Figures 3, 4 and 5. [file 1471-2164-12-639-S14.JPEG]
